# Grating lobe mitigation on large-pitch arrays using null subtraction imaging

**DOI:** 10.1016/j.ultras.2024.107302

**Published:** 2024-03-24

**Authors:** Mick Gardner, Rita J. Miller, Michael L. Oelze

**Affiliations:** Electrical and Computer Engineering, University of Illinois at Urbana-Champaign, 306 N Wright St, Urbana, IL, 61801, USA

**Keywords:** Null Subtraction Imaging, Beamforming, Apodization, Sparse array, f-number, Grating lobes

## Abstract

Null Subtraction Imaging (NSI) is a novel beamforming technique that can produce B-mode images resulting in high spatial resolution and low computational cost compared to other beamforming techniques. Previous work has demonstrated that in addition to a beam pattern with a narrow main lobe and low side lobes, NSI can also reduce or mitigate grating lobes, which can appear when the array pitch is larger than one half the wavelength of the transmitted pulse. These grating lobes can result in imaging artifacts that produce clutter and lower contrast. By lowering grating lobe levels, a larger pitch array could be used, which could allow arrays with a larger field of view while maintaining a standard element count. This could have important benefits for specific applications such as ultrasonic abdominal imaging. Experiments were conducted to examine the feasibility of using NSI with large pitch, wide field-of-view arrays. Grating lobe reduction was measured against array pitch, DC offset, and f-number. Experiments were conducted on wire targets and contrast targets in a phantom and results were further verified in vivo by imaging the liver of a rabbit. The results demonstrated that NSI was able to reduce grating lobe brightness by up to 45 dB compared to delay-and-sum (DAS) beamforming when using planewave transmissions, reduce the generalized contrast-to-noise ratio (gCNR) of grating lobe regions from 0.60 to 0.08, and maintain a similar speckle quality to DAS. The gCNR of anechoic regions also improves, increasing from 0.09 to 0.15 on an array with a pitch of 5 wavelengths. Due to significant grating lobe level reduction, NSI shows potential to improve image quality over DAS on a large pitch, wide field-of-view array.

## 1. Introduction

Large aperture arrays are attractive in the field of medical ultrasound because they provide many benefits to image quality. A large aperture creates a wide field-of-view (FOV), which allows for imaging bigger structures and regions of interest. A wide aperture can also provide a smaller f-number, which increases lateral resolution. Large apertures can also maintain high frame rates and more consistent lateral resolution versus depth when compared to swept apertures or sector scans.

Several approaches have been investigated to produce large aperture arrays. In the simplest approach, a large aperture array can be constructed by using a large number of ultrasonic elements. For example, Bottenus et al. simulated an array with 270 × 54 elements [1], and Foiret et al. stitched 3 commercial probes together for a wide array with 384 elements [2]. Increasing the number of elements can provide a larger field of view while still maintaining a small pitch so that grating lobes do not appear. Also, with more elements transmitting, more total acoustic power propagates into the body, increasing the SNR and imaging depth. However, a large number of elements increases the cost of producing the probe and increases data processing time.

In another approach, large virtual arrays were created by stitching together multiple frames from a probe swept laterally [3,4]. However, methods involving swept apertures can suffer from motion artifacts, which appear as breaks in the image where consecutive frames were stitched together. In addition, swept apertures offer no resolution improvement with their wide FOV because they use standard size transducers. Lastly, the data acquisition time is increased due to the need to acquire multiple frames from different lateral locations.

In yet another approach, diverging waves were used to create a larger field of view without transducer motion or adding extra elements [5,6]. These methods also are able to maintain a relatively high resolution at greater depths than typical plane-wave imaging, because the compounded area does not decrease at greater depths with diverging waves like it does with steered plane waves. However, diverging waves can trade off resolution for potential frame rates depending on the amount of compounding desired.

Rather than increasing the element count while maintaining a small pitch, the width of the array could be increased by increasing the pitch while maintaining a standard element count. Extending the field of view in this manner would not require as many transmissions for a single image, and thus not suffer from motion artifacts to the same degree as diverging wave techniques or swept apertures. The larger width would also decrease the f-number, which improves lateral resolution. Lastly, maintaining a standard number of elements keeps the cost and data processing time comparable to commercially available probes.

However, one major roadblock to extending the pitch of arrays is the introduction of grating lobes. Grating lobes can appear in beam patterns of electronically steered arrays when the pitch is higher than one half the wavelength of the operating frequency of the array. Grating lobes result in unwanted artifacts and clutter that could obstruct important details, as well as reduce the contrast of regions in the image [7]. Therefore, linear probes are usually built with a pitch close to one wavelength. With such a pitch, no grating lobes would be present in the field of view when performing linear sequential scanning. However, with steering of the array, such as with plane wave compound imaging, grating lobes can appear in the field of view unless the pitch is reduced. As a result of the image degradation from grating lobes, increasing the pitch size has not previously been regarded as a solution for wide FOV imaging.

Several techniques have been explored to mitigate grating lobes arising from large pitch to wavelength ratios. One set of techniques is to build specialized probes with reduced pitch, or in some cases, non-uniform pitch [8]. Many phased arrays exist with uniform pitch sizes around one half the wavelength to prevent grating lobes when electronically steering the array. The increased element count necessary for producing a lower pitch greatly increases the cost and data processing of phased array probes. Non-uniform pitch sizes are generally built by removing select elements from a uniform array. However, these are not standard practice on modern ultrasound systems due to an increase in side lobe levels for every element removed [8].

Alternative techniques to mitigate grating lobes involve signal and image processing techniques. In one approach, amplitude modulated chirp signals have been used to increase the bandwidth of an imaging source and to reduce grating lobe power by up to 10 dB [9]. In another approach, a median filter was used on the pressure distribution of ultrasound tomography data to reduce the effect of grating lobes in tomographic images [10].

An adaptive beamforming technique, called Phase Coherence Factor (PCF) beamforming, was investigated, which calculates a weight for each pixel based on the phase variance of the channel data [11,12]. In this method, a high phase variance will create a small coherence factor to darken pixels that come from incoherent channel data. Due to a short pulse length used for ultrasound imaging, grating lobes will only be phase aligned in a few elements in the receive aperture, and the phases in the rest of the elements will be dispersed and produce a high variance. This variance causes PCF beamforming to darken pixels corresponding to grating lobes while preserving pixels corresponding to main lobes, where every channel is phase aligned. However, this method can often increase the variance of speckle in medical ultrasound images, which can lower the contrast-to-noise ratio. Several methods are being researched to mitigate this effect of PCF [13].

As a final example, a method was developed that used a lateral transducer shift of one half the array pitch between consecutive frames to create a virtual array with effectively double the elements at half the original pitch [14]. Because the virtual array had half the pitch of the original array, grating lobes were pushed out to such extreme angles that they no longer appeared in the images. However, this again required a physical translation of the transducer which reduced the frame rate and makes it susceptible to tissue motion.

In this study, we examine the ability of Null Subtraction Imaging (NSI) to reduce and remove grating lobes in linear arrays with large pitch [15]. NSI was introduced recently as a new non-linear beamforming technique which has the capability of reducing grating lobes. The original goal of NSI was to improve lateral resolution in B-mode images while maintaining low side lobes. It was later found that NSI reduced clutter due to grating lobes in rat tumor images when using NSI with plane-wave angular compounding [16]. NSI has also been shown to improve resolution and reduce grating lobes in power Doppler images [17]. The purpose of the research in this paper is to evaluate the ability of NSI to create high-quality images from a large aperture array with a pitch higher than a wavelength.

## 2. Methods

### 2.1. Delay-and-sum implementation

Delay-and-Sum (DAS) beamforming was implemented by establishing a pixel grid, calculating time delays for each pixel location, and summing across an appropriate sub-aperture. Plane-wave data was read from a .mat file saved from a Versonics 128 Vantage (Kirkland, WA) system and reshaped into a 3D matrix of size N×M×L, where N is the number of samples, M is the number of receive channels, and L is the number of plane wave angles. No up-sampling or filtering was performed on the RF data received from the Verasonics system.

The images in this paper were beamformed with pixels at every λ/8 location in both the axial and lateral directions, where λ represents wavelength. Time delays for each pixel were calculated using equations from [18]. The round-trip time from a pixel location ${(x}_{p},\;z_{p})$ to an element i from the transducer is the following:

(1) $$\tau_{i}\left(x_{p},\;z_{p}\right)=\frac{d_{TX}\left(x_{p},\;z_{p}\right)\;+\;d_{RX}\left(x_{p},\;z_{p},\;x_{i}\right)}{c}$$

where $d_{TX}$ and $d_{RX}$ are the transmit and receive times respectively, and $x_{i}$ is the lateral location of element $i.\;d_{TX}$ for a plane-wave transmission is given in [18] as the following:

(2) $$d_{TX}\left(x_{p},z_{p}\right)=\left(sgn(\theta )\frac{L}{2}-x_{p}\right)\;sin(\theta )+z_{p}\;cos(\theta )$$

where θ is the plane-wave steering angle, L is the total aperture size, and sgn() is the signum function. $d_{RX}$ from a pixel location to an element of the transducer aperture is then given in [18] as the following:

(3) $$d_{RX}\left(x_{p},z_{p},x_{i}\right)=\sqrt{{\left(x_{i}-x_{p}\right)}^{2}+z_{p}^{2}}.$$

Once the time delays were calculated, they were converted to sample delays by simply dividing by the sample rate and rounding. No interpolation was done to estimate fractional sample delays.

Receive beamforming was done on a sliding sub-aperture, such that for a given pixel location and a sub-aperture size of M, M/2 elements to the left of the pixel and M/2 elements to the right of the pixel were summed together after the appropriate time delays were applied. A rectangular apodization was used for receive beamforming in the DAS images shown throughout the paper.

Once every pixel of every steering angle was beamformed, steering angles were summed to implement angular compounding. Envelope detection was then performed using IQ demodulation, where the compounded signal was multiplied by sine and cosine functions at the center frequency of the transmitted pulse to get in-phase (I) and quadrature (Q) components. I and Q components were then individually low-pass filtered using coefficients resulting from MATLAB’s designfilt() function with the following parameters: designfilt(‘lowpassfir’,’FilterOrder’,4, ‘PassbandFrequency’,0.4,’StopbandFrequency’,0.7). The envelope was then calculated as env=|I+jQ|.

### 2.2. Null subtraction imaging

NSI is a beamforming technique that images with nulls in an array beam pattern to achieve a high lateral resolution while maintaining low side lobes. NSI consists of beamforming on receive with three apodizations in parallel: one with zero mean, and two that are an offset version of the zero mean apodization. The zero-mean apodization consists of weightings with an equal number of +1 and −1 on either half of the receive sub-aperture. This apodization is given by the following equation [16]:

(4) $$A_{ZM}(i)=\left\{\begin{array}{ll} 1, & 1\leq i<\frac{N}{2}\\ -1, & \frac{N}{2}\leq i\leq N \end{array}\right.$$

where N is the number of receive elements. This apodization creates a null at 0° or broadside. The offset versions of the zero-mean apodization are given by the following formula [16]:

(5) $$A_{DC1}(i)=A_{ZM}(i)+dc$$

where dc is a constant amount of offset, referred to as the DC offset. The DC offset is a tuneable parameter of NSI, typically in the range 0.1–1. This offset has the effect of bridging the null created from the zero-mean apodization, while maintaining similar side lobes. Two DC apodizations are needed because the offset changes the absolute values of the weights so they are no longer symmetric (e.g. with a DC offset of 0.5, +1/−1 becomes +1.5/−0.5). This creates an uneven beam pattern where more energy is concentrated to one side of the array [15]. Therefore, the second DC apodization is a mirror of the first, i.e. $A_{DC2}(i)=A_{DC1}(N-i)$, where N is the number of receive elements. After beamforming is done with the three apodizations, the envelopes are taken and applied in the following equation [16]:

(6) $$E_{NSI}=\frac{E_{DC1}\;+\;E_{DC2}}{2}-E_{ZM}$$

where $E_{NSI}$ is the final NSI envelope image, $E_{DC1}$ and $E_{DC2}$ are the DC offset envelope images, and $E_{ZM}$ is the zero-mean envelope image. Because Eq. (6) takes place after envelope detection, which is a non-linear operation, NSI is a non-linear processing technique.

NSI was implemented by performing DAS beamforming three times as described in Section 2.1 on copies of the same RF data with the three receive apodizations ($A_{ZM},A_{DC1},A_{DC2}$). To use NSI in conjunction with plane-wave angular compounding, the three NSI apodizations are applied individually to each steering angle as well. Then, the resulting signals are summed over the angle dimension, envelope detected, and applied in Eq. (6) to produce the final NSI envelope. This method is known as “coherent NSI” or “C-NSI” [16]. All other DAS parameters such as the pixel locations, receive sub-aperture size, and IQ demodulation/low-pass filter for envelope detection, are the same for NSI, meaning the only difference is the receive apodizations.

### 2.3. Image quality assessment

In this study, grating lobe reduction and contrast improvement resulting from NSI were assessed against a changing pitch size, DC offset, and f-number. Image quality was further assessed in vivo by imaging a rabbit abdomen. DAS and NSI images were reconstructed for each of these experiments and compared qualitatively. Additionally, two quantitative metrics for images were estimated in each experiment to quantify the amount of grating lobe reduction and contrast improvement resulting from NSI. The first metric was the generalized contrast-to-noise ratio (gCNR), and the second was the brightness reduction of a grating lobe relative to its corresponding main lobe.

The gCNR is given by the following formula [19]:

(7) $$gCNR=C_{0}^{-\frac{C_{0}}{C_{0}-1}}-C_{0}^{-\frac{1}{C_{0}-1}}.$$

In this equation, $C_{0}$ is the contrast of some region given by $C_{0}=\mu_{i}/\mu_{o}$, where $\mu_{i}$ is the average envelope level inside some region of interest, and $\mu_{o}$ is average envelope level of the background. The gCNR was estimated for grating lobe regions and anechoic cysts in order to quantify the brightness of these regions relative to the background.

gCNR is used for two reasons. First, because NSI involves summation of receive beams after envelope detection, it is possible that NSI changes the dynamic range of the image. It has also been demonstrated that NSI increases the speckle variance, which causes the contrast-to-noise ratio (CNR) of images to suffer, even for images that become qualitatively more clear [16]. Thus, gCNR is used to base image quality on the probability of detection, which is resilient against changes in dynamic range and speckle variance [19]. The ideal gCNR of a grating lobe region would be zero, as that would represent the case where the grating lobe was completely mitigated. Likewise, the ideal gCNR of an anechoic cyst would be 1, as that would represent perfect detectability of that region.

In addition to gCNR, the brightness reduction of a grating lobe was also estimated using the following equation:

(8) $$\mathrm{reduction}=\left(\mu_{\mathrm{main},DAS}-\mu_{\mathrm{grating}\;,DAS}\right)\;\;-\left(\mu_{\mathrm{main}\;,NSI}-\mu_{\mathrm{grating}\;,NSI}\right)$$

where in this case, μ represents the average pixel brightness in dB of a region. For example, $\mu_{\mathrm{main},\mathrm{DAS}\;}$ is the average brightness of a main lobe for delay-and-sum, while $\mu_{\mathrm{grating},NSI}$ is the average brightness of a grating lobe region in NSI. This metric represents the reduction in grating lobe brightness relative to their corresponding main lobes that NSI achieved over DAS.

### 2.4. Experiments

#### 2.4.1. Grating lobe reduction versus array pitch

Datasets of wire targets and contrast targets embedded in a CIRS Model ATS 539 general purpose phantom were acquired with both L14–5/38 and L14–5/60 probes connected to a Versonics system. These probes both have 128 elements, but a different pitch and element size. The L14–5/38 has a pitch of 0.3 mm and an element size of 0.27 mm, while the L14–5/60 has a pitch of 0.472 mm and an element size of 0.447 mm. On each probe, the pitch relative to a wavelength was varied by changing the frequency of the excitation pulse. Frequencies of 5 MHz, 7.81 MHz, and 10 MHz were used. Changing the excitation frequency effectively changes the pitch in the sense that the ratio between element spacing and wavelength will be different. Further pitch variation was achieved by using channel decimation of two on both transmit and receive for some data acquisitions, effectively doubling the original pitch. In total, 8 distinct pitch sizes relative to a wavelength were tested. The pitch sizes and corresponding settings are shown in Table 1.

**Fig. 1.**
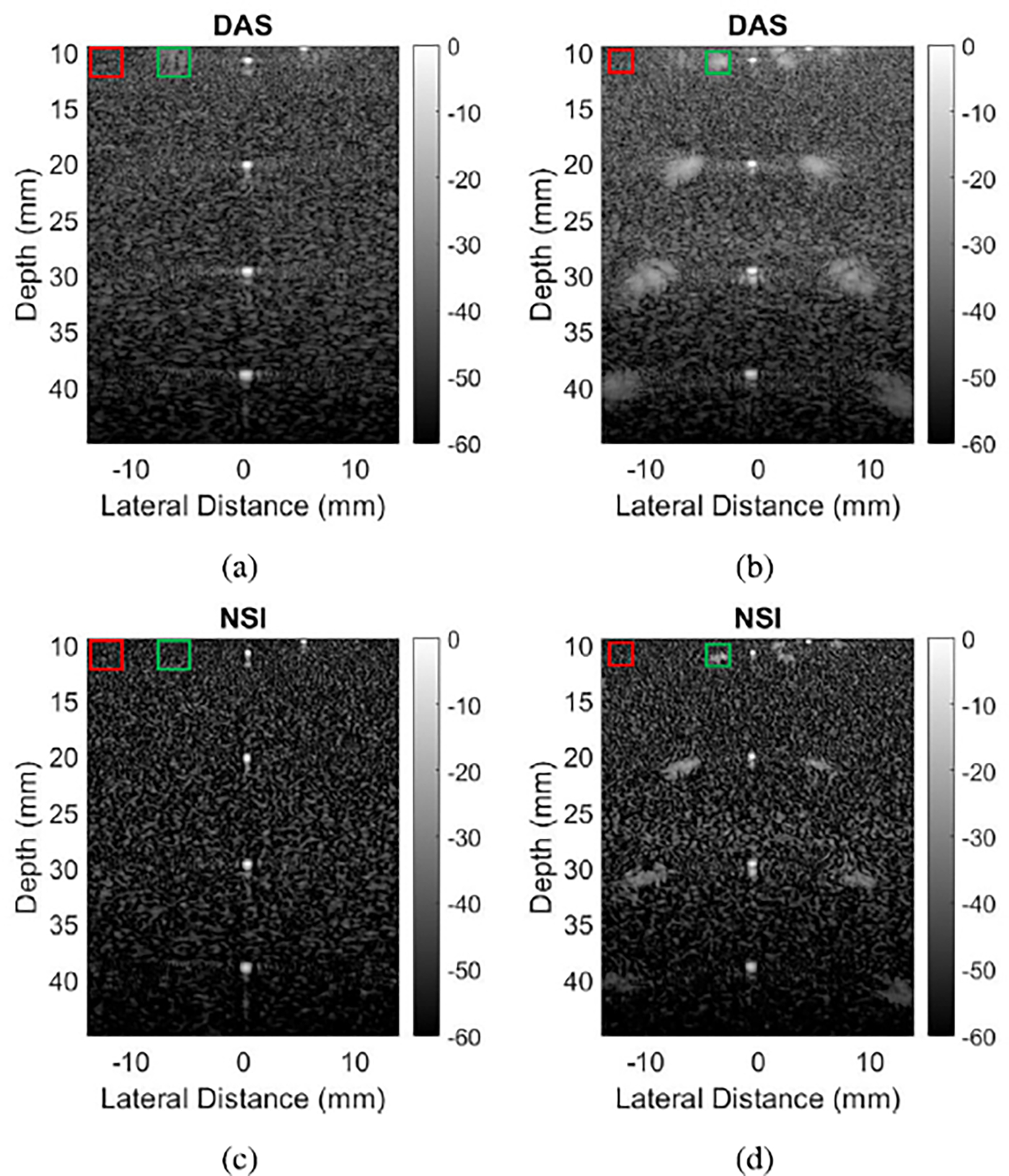
B-mode images of wire targets reconstructed with DAS and NSI. (a) DAS with 2.5λ pitch, resulting gCNR −0.43. (b) DAS with 5λ pitch, resulting gCNR −0.69. (c) NSI with 2.5λ pitch, resulting gCNR −0.18. (d) NSI with 5λ pitch, resulting gCNR −0.62. Green boxes represent “in” regions for Eq. (7), and red boxes “out” regions.

**Fig. 2.**
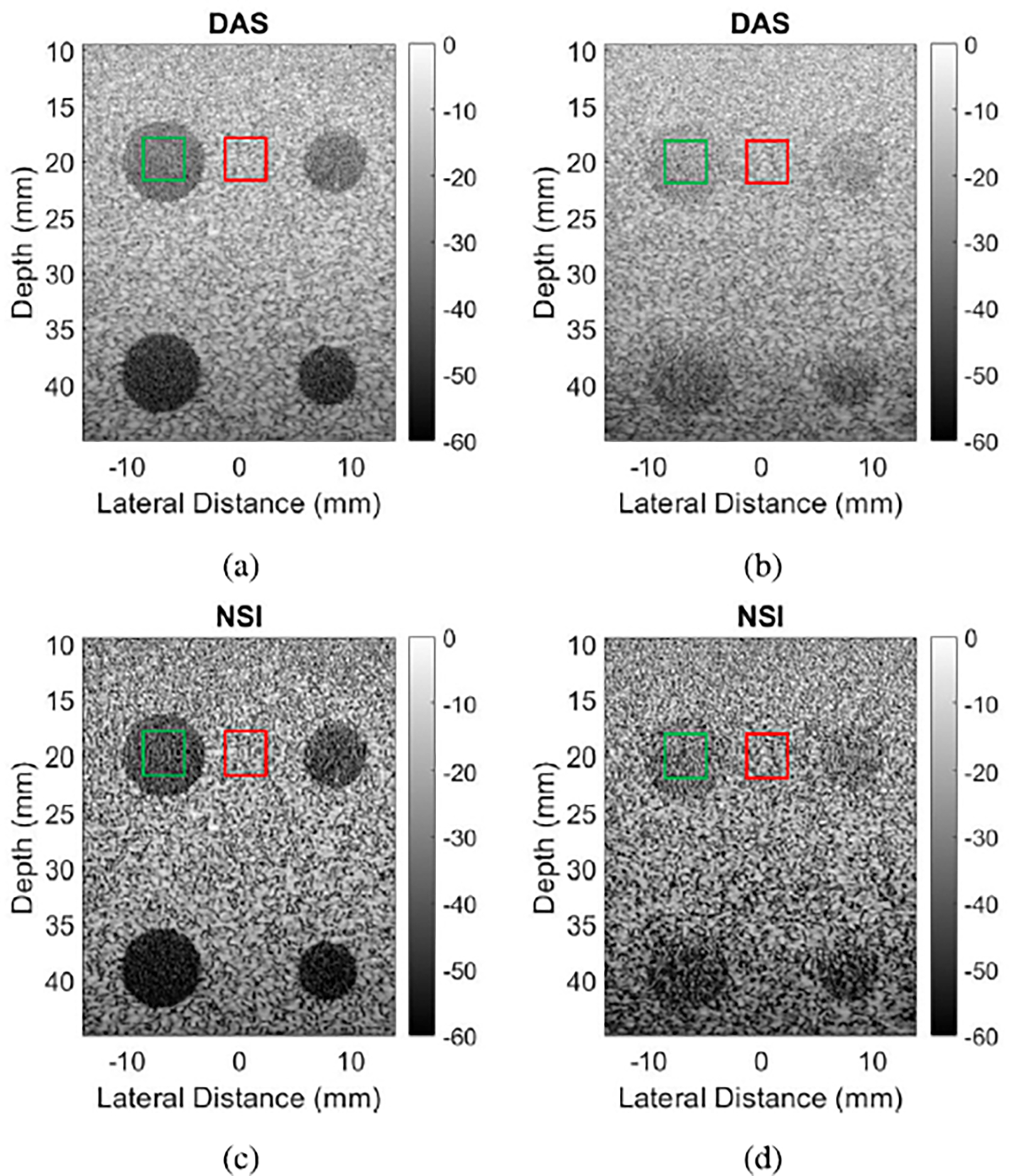
B-mode images of anechoic targets reconstructed with DAS and NSI. (a) DAS with 2.5λ pitch, resulting gCNR 0.52. (b) DAS with 3.2λ pitch, resulting gCNR 0.19. (c) NSI with 2.5λ pitch, resulting gCNR 0.62. (d) NSI with 3.2λ pitch, resulting gCNR 0.27. Green boxes represent “in” regions for Eq. (7), and red boxes “out” regions.

For angular compounding, 11 steering angles over the range of −10° to +10° were acquired for the wire targets, while 37 angles over the range of −18° to +18° were acquired for contrast targets. The receive subaperture size was 32 elements for undecimated datasets, and 16 elements for decimated datasets. For comparison, images were reconstructed for each probe using both DAS and NSI with a DC offset of 1. gCNR and brightness reduction were estimated from grating lobes observed on the left side of a wire located at 10 mm depth, shown by the boxes in Fig. 1. This wire target was the only one of the four that produced grating lobes inside the image at all pitch sizes. gCNR was also estimated for an anechoic region with a radius of 3 mm located with its center at 20 mm depth, shown by the boxes in Fig. 2.

#### 2.4.2. Grating lobe reduction versus DC offset

To test DC offsets, wire targets and contrast targets from the same phantom were imaged using an L14–5/60 probe operating at 7.81 MHz with channel decimation of two. This creates a pitch size of about 5 wavelengths (see Table 1). The dataset was acquired using plane-wave imaging, with 11 steering angles over the range of −10° to +10° for the wire targets, and 37 steering angles over the range of −18° to +18° for the contrast targets.

A total of 13 DC offsets were tested in this experiment, chosen to be evenly spaced on a log scale between 0.01 and 10. With this set of DC values, broad trends over several orders of magnitude could be observed. gCNR and brightness reduction were measured for every DC value based on the right grating lobe from a wire at 20 mm depth, shown by the boxes in Fig. 4. This target was used instead of the one at 10 mm because at a 5λ pitch, grating lobes are visible at a depth of 20 mm and they cover a larger region which makes placement of the region of interest simpler. Additionally, gCNR was measured for an anechoic region with a radius of 3 mm located at 20 mm depth, shown by the boxes in Fig. 5.

As an added step for this experiment, the speckle signal-to-noise ratio (sSNR) was also estimated for the background of each image. The sSNR of an ultrasound image is given by $sSNR=|\overset{‾}{A}|/\sqrt{var(A)}$, where A is the envelope level in some region of interest, $\overset{‾}{A}$ is the mean and var(A) is the variance of the envelope in that region. For fully developed speckle, the sSNR should be around 1.91 [20]. Anything lower suggests underdeveloped speckle. Because it has been observed that NSI both darkens and increases the variance of the speckle depending on the DC value [15,16], this metric was included to quantify those changes.

#### 2.4.3. Grating lobe reduction versus F-number

This experiment used the same plane-wave datasets as the DC offset experiment detailed in Section 2.4.2, which used the L14–5/60 probe at a frequency of 7.81 MHz with channel decimation of 2, resulting in a pitch size of 5 wavelengths. For this experiment, the DC offset was held constant at 1 while the receive sub-aperture size changed to vary the f-number. The f-number is given by $f^{\#}=D/A$, where $f^{\#}$ is the f-number, D is the imaging depth, and A is the receive sub-aperture size. The receive sub-aperture sizes used were 4, 8, 16, and 32 elements after channel decimation, which correspond to sub-aperture widths of 2.98 mm, 6.96 mm, 14.91 mm, 30.81 mm, respectively. Grating lobe gCNR and brightness reduction were estimated for grating lobes observed on the left side of wire targets at depths of 10 mm, 20 mm, 30 mm, and 40 mm. The varied receive sub-aperture sizes and varied target depths resulted in a range of f-numbers from 0.32–13.42 being tested for wire targets. Additionally, gCNR was measured for two anechoic regions at depths of 20 mm and 40 mm, using the same four sub-aperture sizes. This resulted in a range of f-numbers from 0.65–13.42 being tested for anechoic regions. Table 2 shows the exact setup for each f-number that was tested.

#### 2.4.4. Rabbit abdomen

Animal experiments were approved by the Institutional Animal Care and Use Committee at the University of Illinois at Urbana-Champaign. The liver of a New Zealand White rabbit was imaged in vivo using an L14–5/60 probe at 7.81 MHz. The rabbit was anesthetized using isoflurane and the fur on the abdomen was shaved. Then, the liver was imaged transabdominally. Datasets were collected both with and without channel decimation (pitches of 5λ and 2.5λ respectively). A receive sub-aperture size of 32 elements was used for undecimated datasets, and 16 elements for decimated datasets. These correspond to f-numbers of 2.01 for a depth of 30 mm (approximate depth of the liver). For these planewave datasets, a total of 31 angles in the range −15° to +15° were collected.

## 3. Results

### 3.1. Grating lobe reduction versus array pitch

Fig. 1 shows example B-mode images of wire targets reconstructed from the L14–5/60 probe, the left column without channel decimation and the right column with channel decimation of two, resulting in pitch sizes of 2.5λ and 5λ respectively. Grating lobe artifacts are observed on either side of the wire at 10 mm in Fig. 1(a), and they are especially apparent on either side of every wire in Fig. 1(b). The same datasets processed with NSI show significant grating lobe reduction, to the point that there is no visible clutter in Fig. 1(c). However, Fig. 1(d) still has visible clutter despite the grating lobe reduction from NSI.

Fig. 2 shows example B-mode images of contrast targets, where the left column was taken with a pitch of 2.5λ and the right column with a pitch of 3.2λ. It can be observed that for both pitch sizes, the NSI versions shown in Figs. 2(c) and 2(d) have darker anechoic regions compared to the corresponding DAS images shown in Figs. 2(a) and 2(b).

**Fig. 3.**
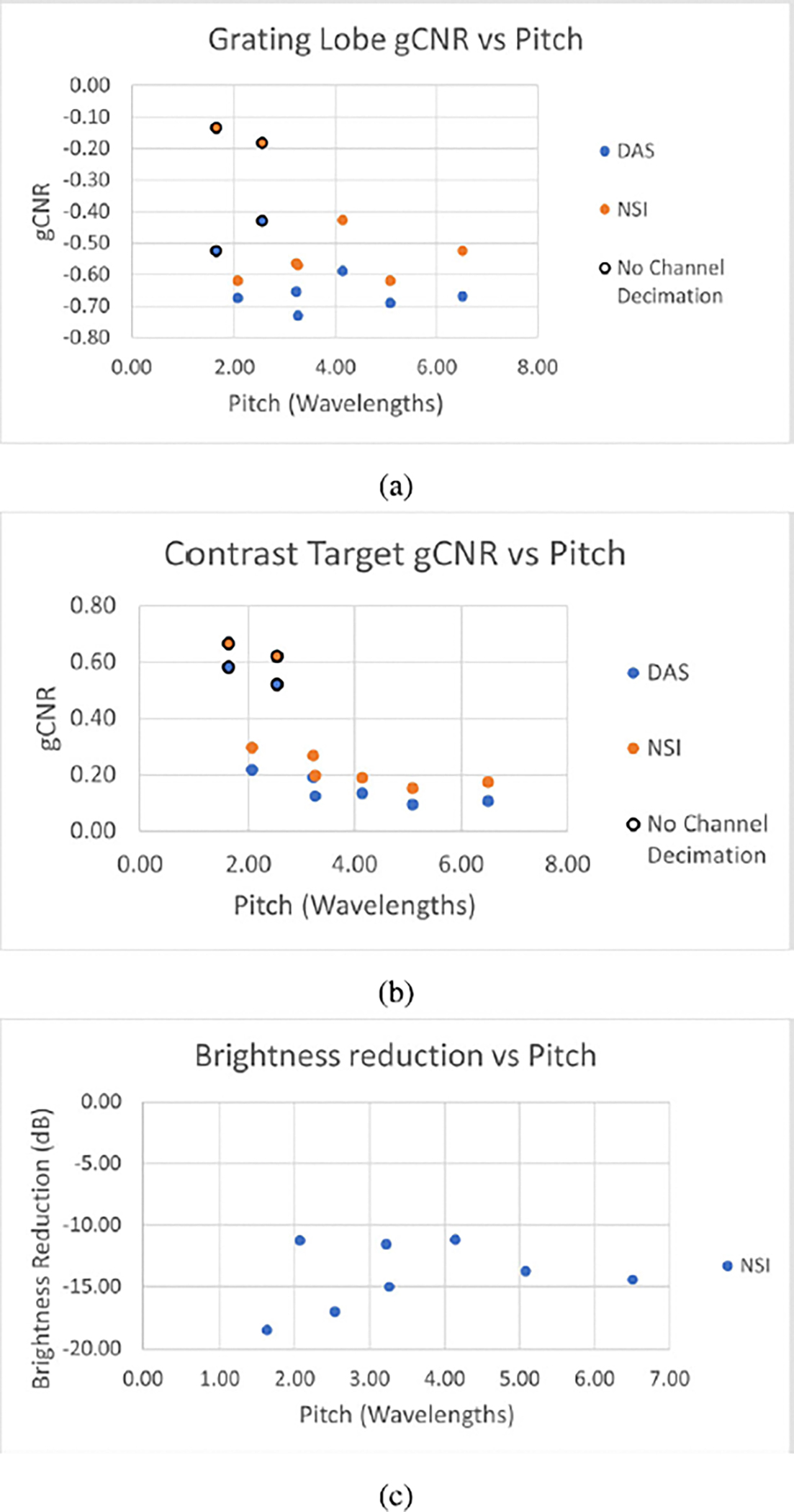
Scatter plots of (a) gCNR of grating lobes, (b) gCNR of anechoic targets, and (c) grating lobe brightness reduction against array pitch.

Fig. 3 shows scatter plots of gCNR estimates and brightness reduction estimates of grating lobes taken at all the pitch sizes, as well as the gCNR of the contrast regions. Note that gCNR is negative for grating lobes, because grating lobes are brighter than the background, while gCNR is positive for anechoic targets, because these targets are darker than the background [19]. Fig. 3(a) shows gCNR values of grating lobes which are consistently closer to their target value of zero for NSI than they are for DAS. Also, the two data points at pitch sizes of 1.63λ and 2.54λ show much greater improvements with NSI than the rest of the data points. Next, Fig. 3(b) shows gCNR values of anechoic targets which are consistently higher for NSI than DAS, though still far from their ideal value of 1 in most cases. In both Figs. 3(a) and 3(b), black outlines mark those data points which were not made with channel decimation. Lastly, the brightness reduction of grating lobes relative to their corresponding main lobe is consistently around 10−15 dB across all pitch sizes, as shown in Fig. 3(c).

### 3.2. Grating lobe reduction versus DC offset

**Fig. 4.**
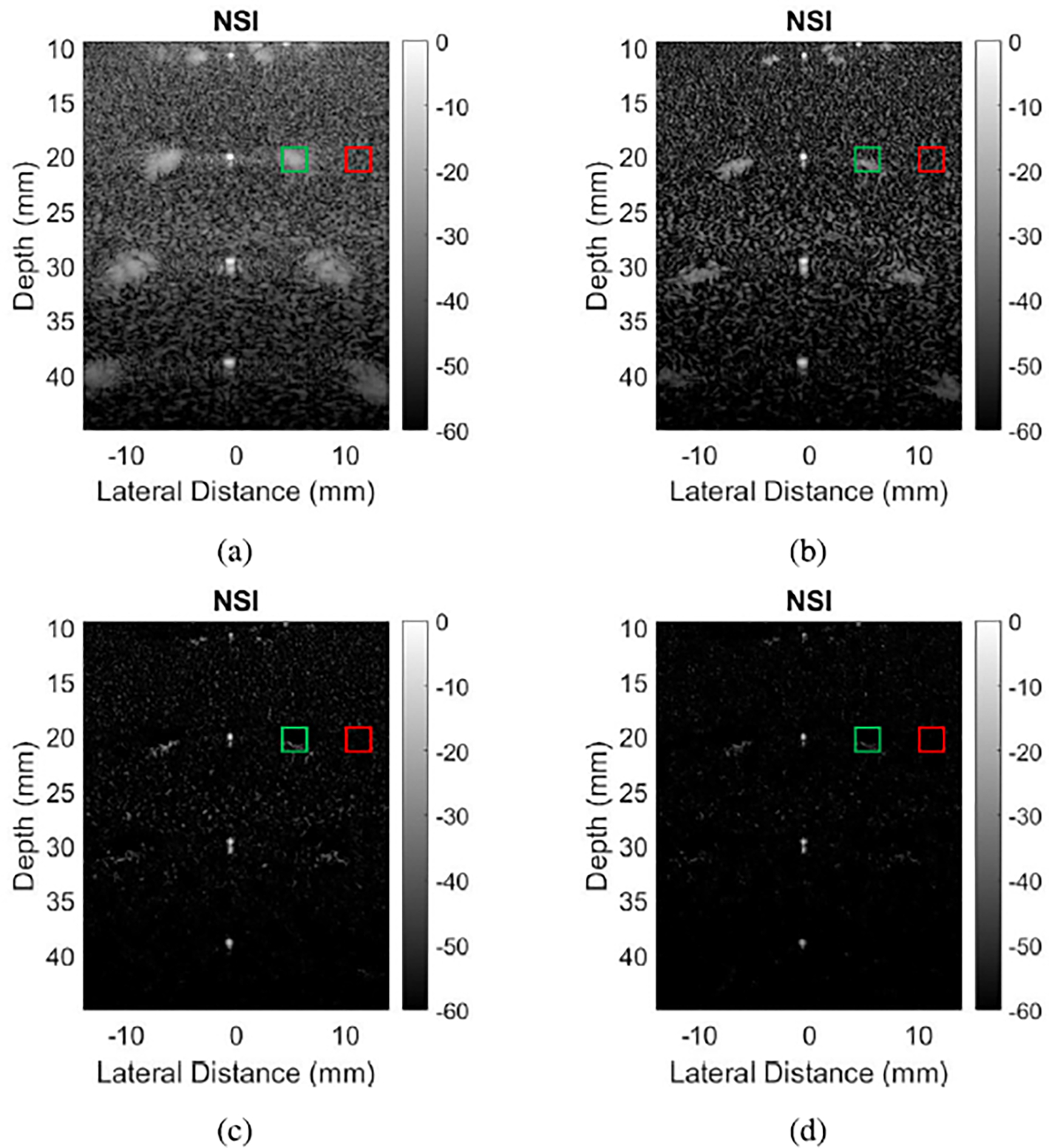
Comparison of wire targets imaged with DC offsets of (a) 10, (b) 1, (c) 0.1, and (d) 0.01. All datasets came from a 5λ pitch. Green boxes represent “in” regions for Eq. (7), and red boxes “out” regions. Red boxes were also used as the ROI for sSNR. The resulting gCNR values for those regions were (a) −0.62, (b) −0.36, (c) −0.09, and (d) −0.07.

**Fig. 5.**
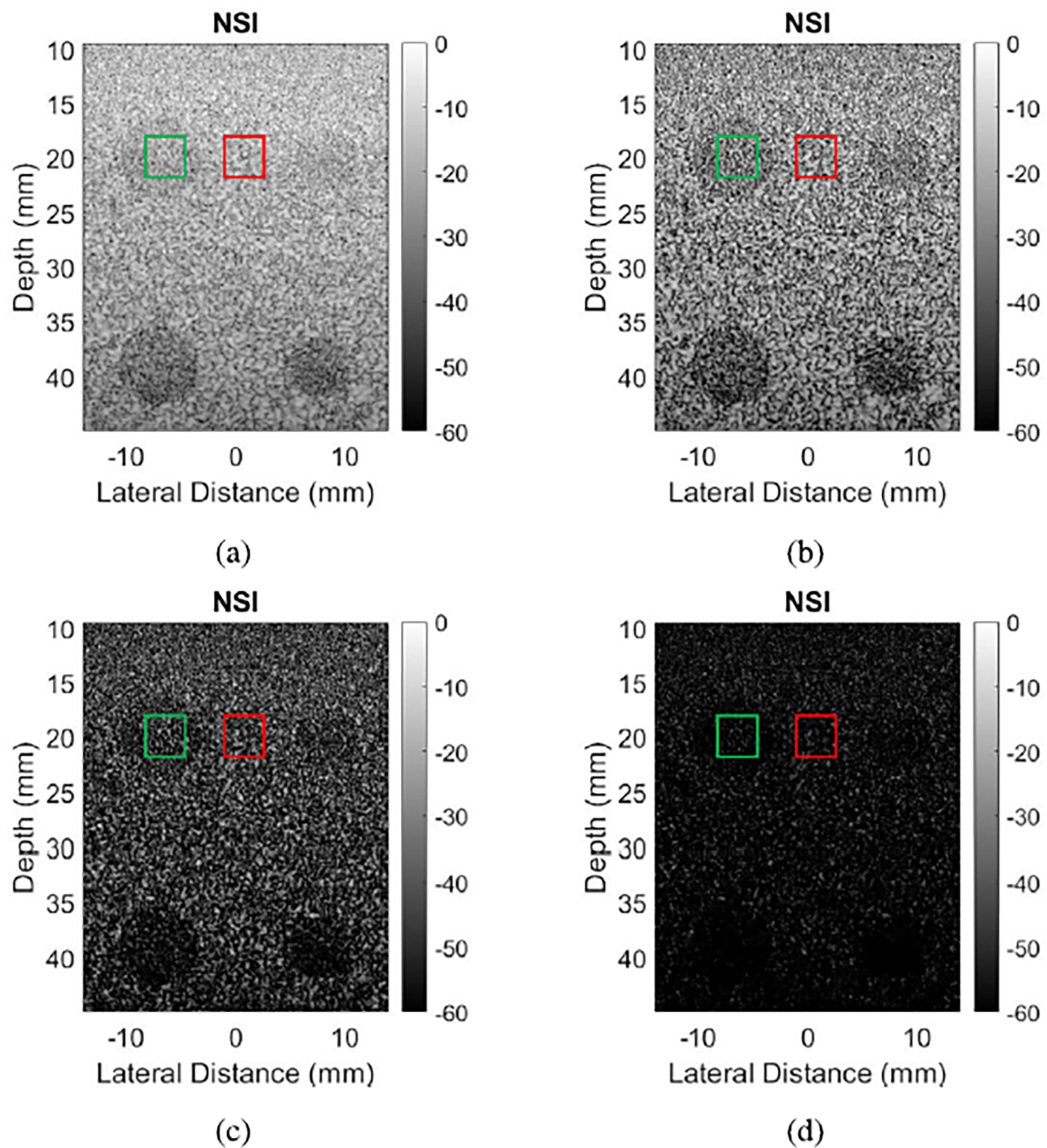
Comparison of anechoic regions beamformed with DC offsets of (a) 10, (b) 1, (c) 0.1, and (d) 0.01. All datasets came from a 5λ pitch. Green boxes represent “in” regions for Eq. (7), and red boxes “out” regions. Red boxes were also used as the ROI for sSNR. The resulting gCNR values for those regions were (a) 0.10, (b) 0.15, (c) 0.17, and (d) 0.17.

Fig. 4 shows example B-mode images of wire targets from NSI using various DC offsets when the pitch was equal to 5λ. Likewise, Fig. 5 shows example B-mode images of contrast targets. In both cases, images darkened and exhibited more grating lobe suppression as the DC offset decreased. At one extreme, the images with a DC offset of 10 in Figs. 4(a) and 5(a) were very similar in appearance to the corresponding DAS images in Figs. 1(b) and 2(b). This similarity is expected for DC offsets much greater than 1 because the DC offset apodizations become closer to a rectangular apodization as the DC offset grows. At the other extreme, the DC offset of 0.01 created images where most grating lobes and speckle were suppressed, creating nearly all black images as observed in Figs. 4(d) and 5(d).

**Fig. 6.**
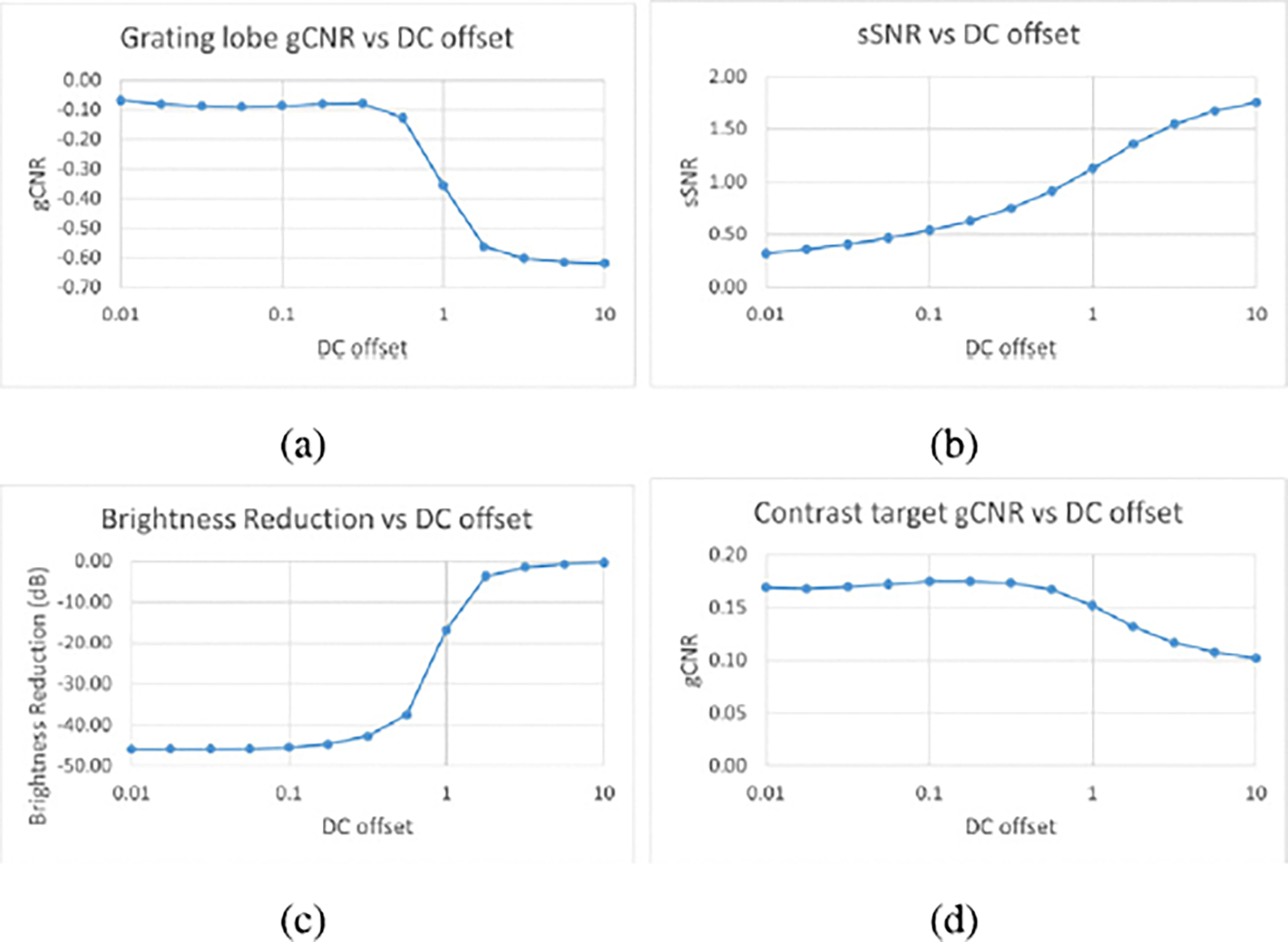
Scatter plots of (a) grating lobe gCNR values, (b) sSNR values, (c) grating lobe brightness reduction values, and (d) anechoic target gCNR values, all for DC values between 0.01 and 10.

The grating lobe gCNR and brightness reduction estimates, as well as gCNR estimates for anechoic targets, for the various DC offsets are shown in Fig. 6. Fig. 6(a) shows the gCNR results on wire targets for the 13 DC values that were tested. The gCNR for grating lobes moved closer to zero with a decreasing DC offset, reached a peak around a DC value of 0.3, then decreased and tapered off with DC offsets lower than 0.3, with resulting gCNR values of around −0.08.

Fig. 6(b) shows the sSNR for the wire target images across the 13 DC offsets. This chart shows a steady decrease in sSNR as the DC offset decreases from 10 to 0.01, which quantifies the darkening of the images in Figs. 4 and 5.

As for brightness levels, Fig. 6(c) shows that the brightness of the grating lobes decreased as the DC offset was lowered from 10 down to about 1. Then, around a DC offset of 1 the brightness level decreased very rapidly until again tapering off at a value of about −45 dB with a DC offset of 0.1 and lower.

Fig. 6(d) shows that gCNR for the contrast targets increases with a decreasing DC offset, until once again reaching a peak at a DC value of around 0.2, similarly to gCNR of grating lobes. For DC offsets greater than 1, as has been explained, the image approaches the appearance of original DAS, so NSI provided less benefit to contrast. For DC offsets much lower than about 0.2, speckle was suppressed so much that contrast began to decrease again.

**Fig. 7.**
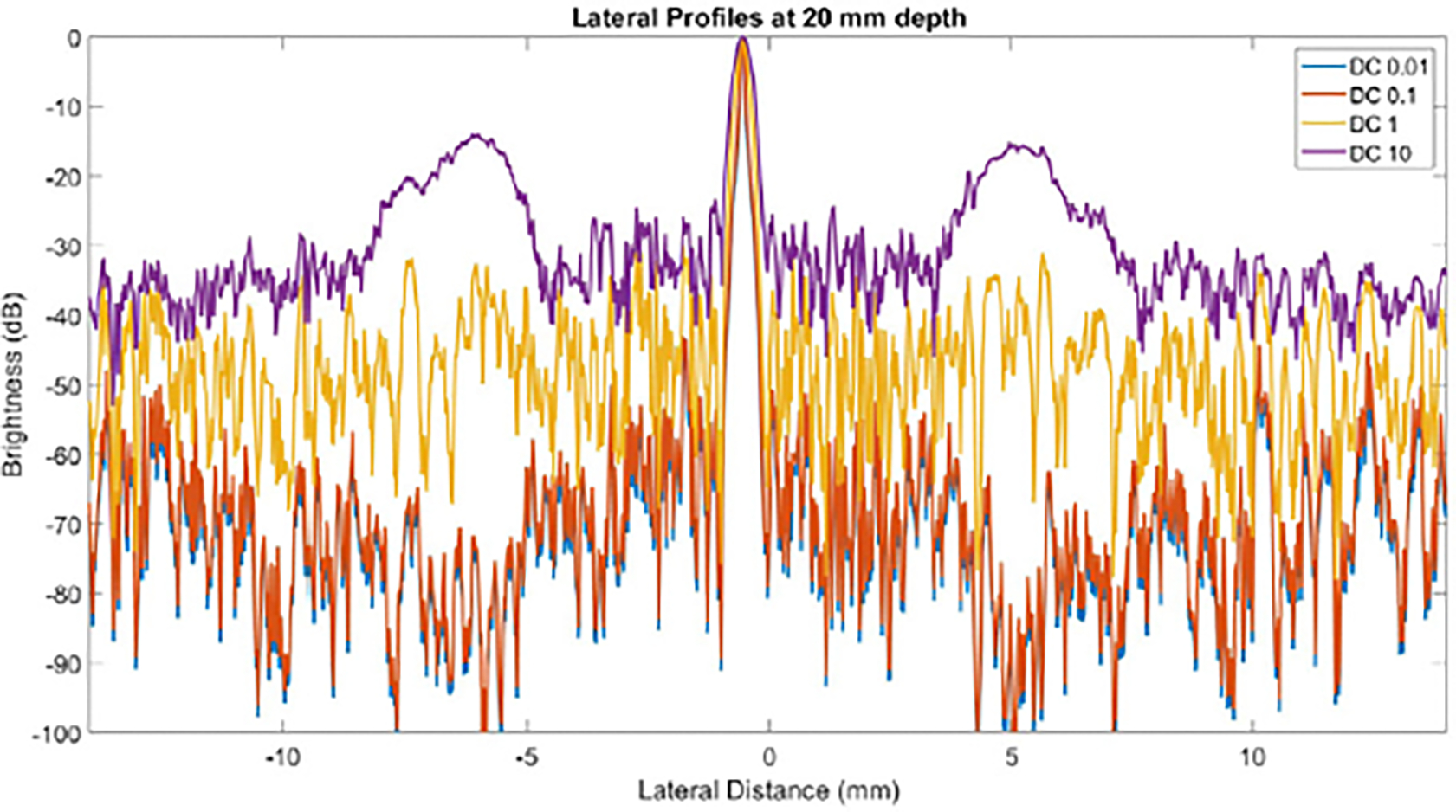
Lateral profiles of a wire target after doing NSI with various DC offsets. Profiles were averaged axially with a width of about 0.5 mm. Profiles for DC 0.1 and 0.01 are so similar that they practically lie on top of each other.

**Fig. 8.**
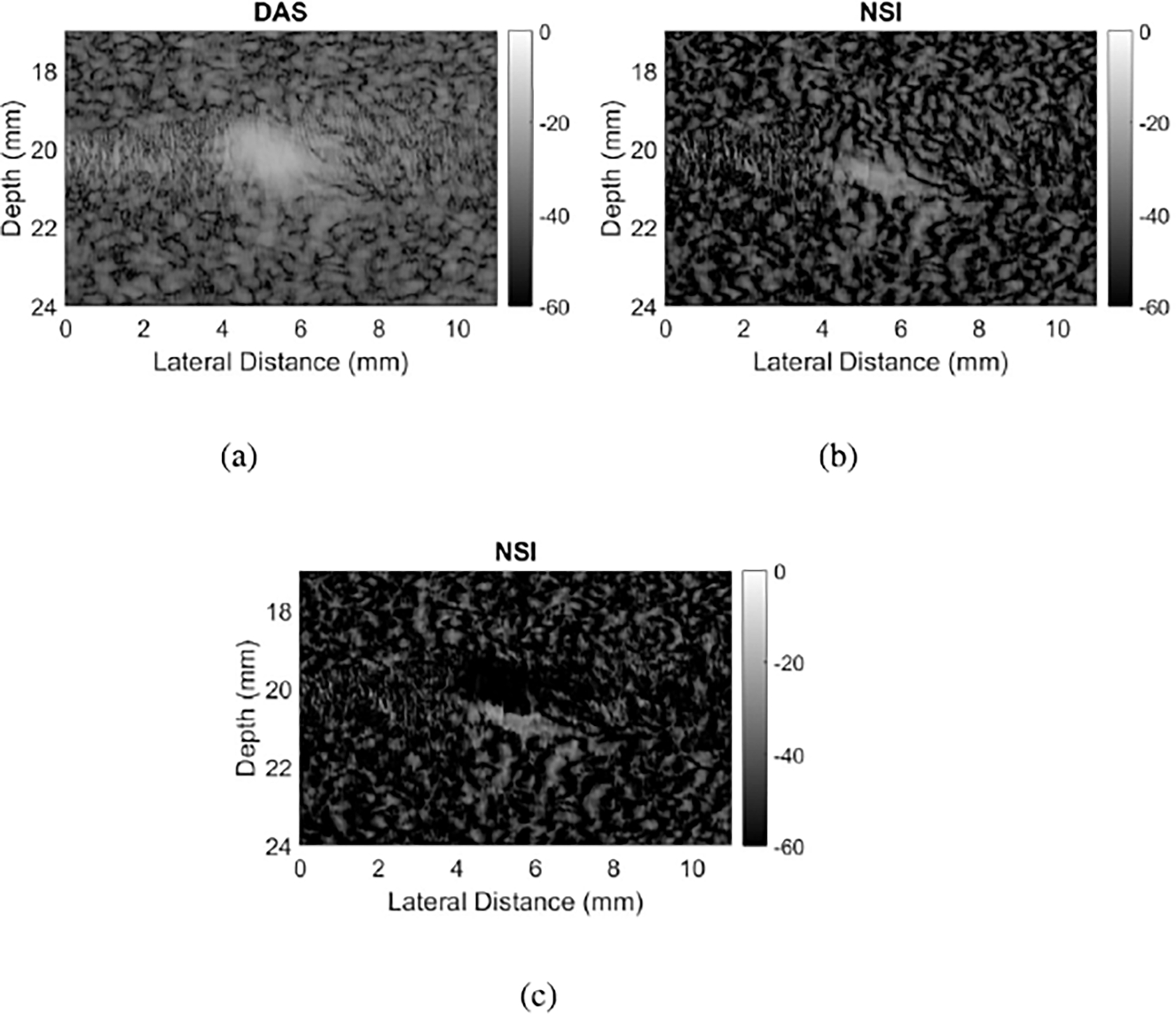
Zoomed in images of a grating lobe at 20 mm, beamformed using (a) DAS, (b) NSI with DC = 1, and (c) NSI with DC = 0.5. Note that in (c), the top portion of the grating lobe region is now darker than surrounding speckle.

Lateral profiles across the wire target at 20 mm depth were plotted in Fig. 7. Each of these profiles represents a lateral cross section of the images around 20 mm, averaged axially with a length of about 0.5 mm. The profile corresponding to a DC offset of 10 shows much higher grating lobes than the other profiles on either side of the main lobe (around 20 dB higher than DC 1). DC offsets of 0.1 and 0.01 show little difference to each other, as their curves basically lie on top of each other. A DC offset of 1 results in grating lobe reduction of around 20 dB compared to DC 10. DC offsets of 0.1 and 0.01 result in grating lobe reductions of more than 40 dB compared to DC 10. However, these larger reductions for the DC offset of 0.1 and 0.01 appear to have inverted the grating lobes, causing them to appear at a lower level than the surrounding speckle. This inversion was observed for all DC offsets less than 1. As an example, Fig. 8 zooms in on one grating lobe for DAS, NSI with DC = 1, and NSI with DC = 0.5. The bright grating lobe region from DAS in Fig. 8(a) is replaced by a dark region from NSI at DC = 0.5 in Fig. 8(c). It appears that NSI with a DC offset of 1 maximally reduces grating lobes while preserving the underlying speckle, as observed in Fig. 8(b).

### 3.3. Grating lobe reduction versus f-number

**Fig. 9.**
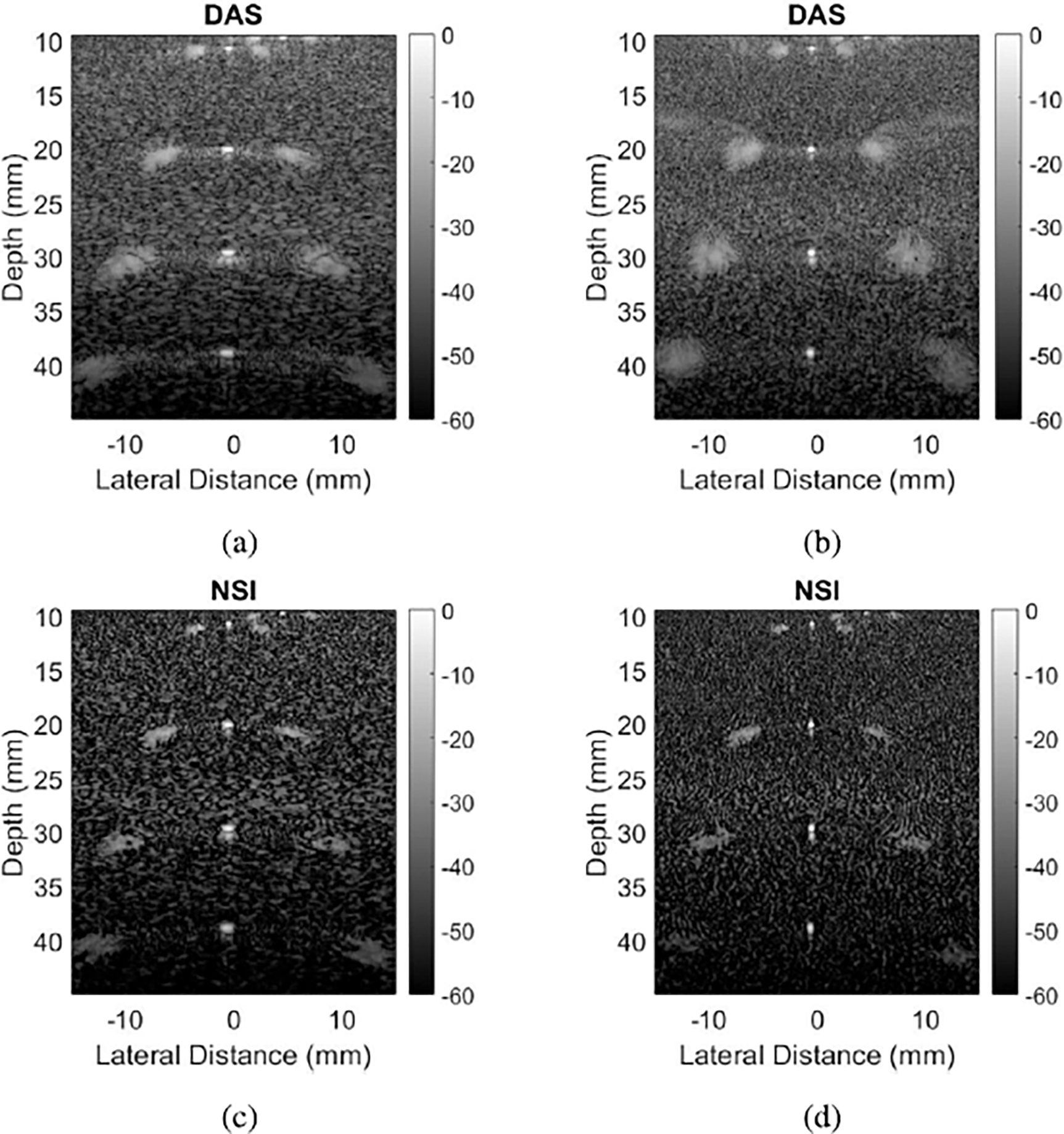
(a–b) DAS and (c–d) NSI images of wire targets with sub-aperture sizes of (a, c) 8 and (b, d) 32 elements. All NSI images were processed with a DC offset of 1.

**Fig. 10.**
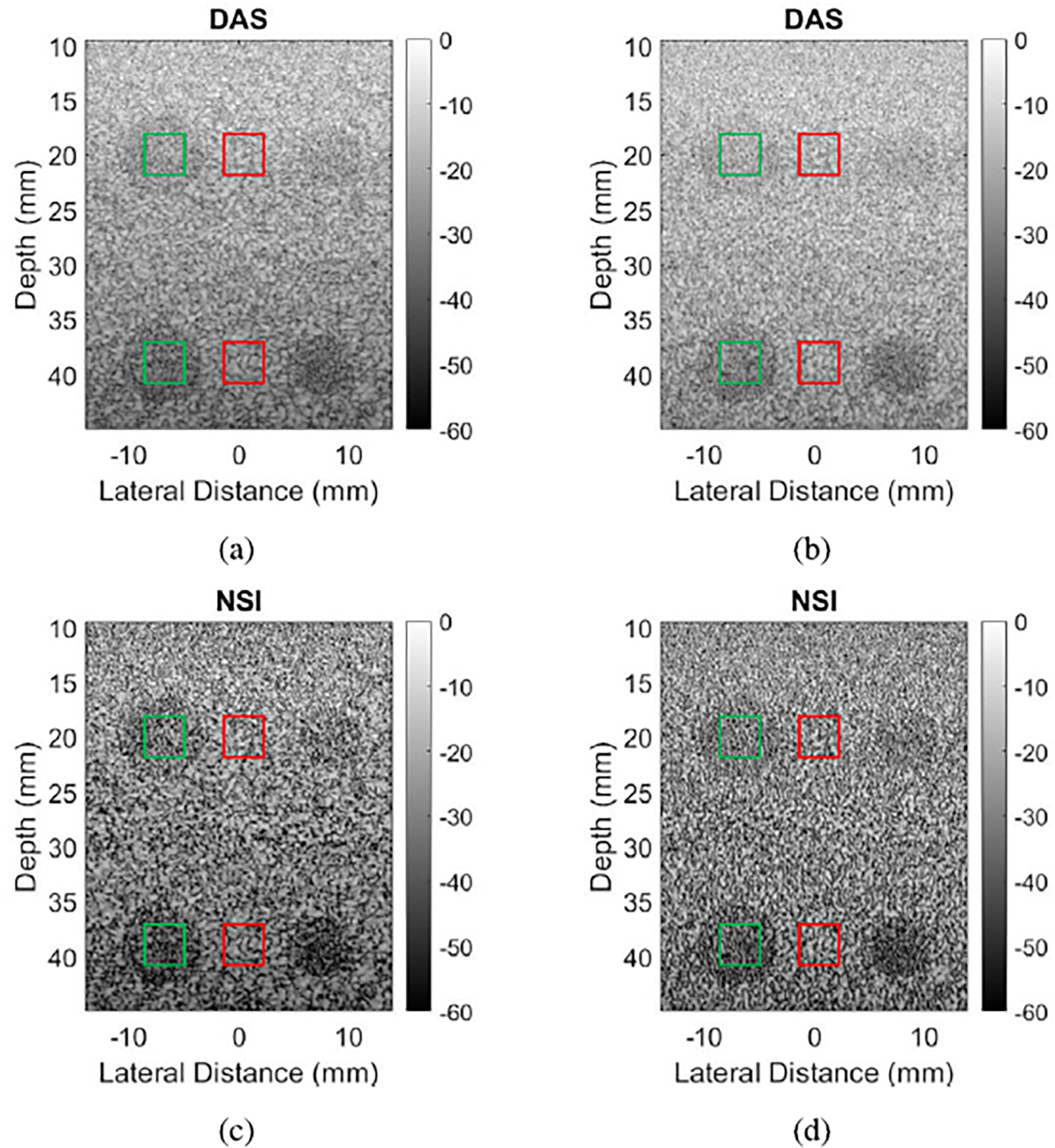
(a–b) DAS and (c–d) NSI images of anechoic targets with sub-aperture sizes of (a, c) 8 and (b, d) 32 elements. All NSI images were processed with a DC offset of 1. Green boxes represent “in” regions for Eq. (7), and red boxes “out” regions. The resulting gCNR values for those regions were (a) 0.15 on top and 0.25 on bottom, (b) 0.07 on top and 0.24 on bottom, (c) 0.20 on top and 0.33 on bottom, (d) 0.12 on top and 0.32 on bottom.

Fig. 9 shows B-mode images of wire targets reconstructed with DAS and NSI at receive sub-aperture sizes of 8 elements in the left column and 32 elements in the right column. While NSI reduces grating lobes in both cases, the reduction is more apparent with the 32-element sub-aperture than the 8-element sub-aperture. Likewise, Fig. 10 shows images of contrast targets at sub-aperture sizes of 8 elements in the left column and 32 elements in the right column. For both f-numbers, the cysts appear slightly darker in the NSI versions than the DAS versions.

**Fig. 11.**
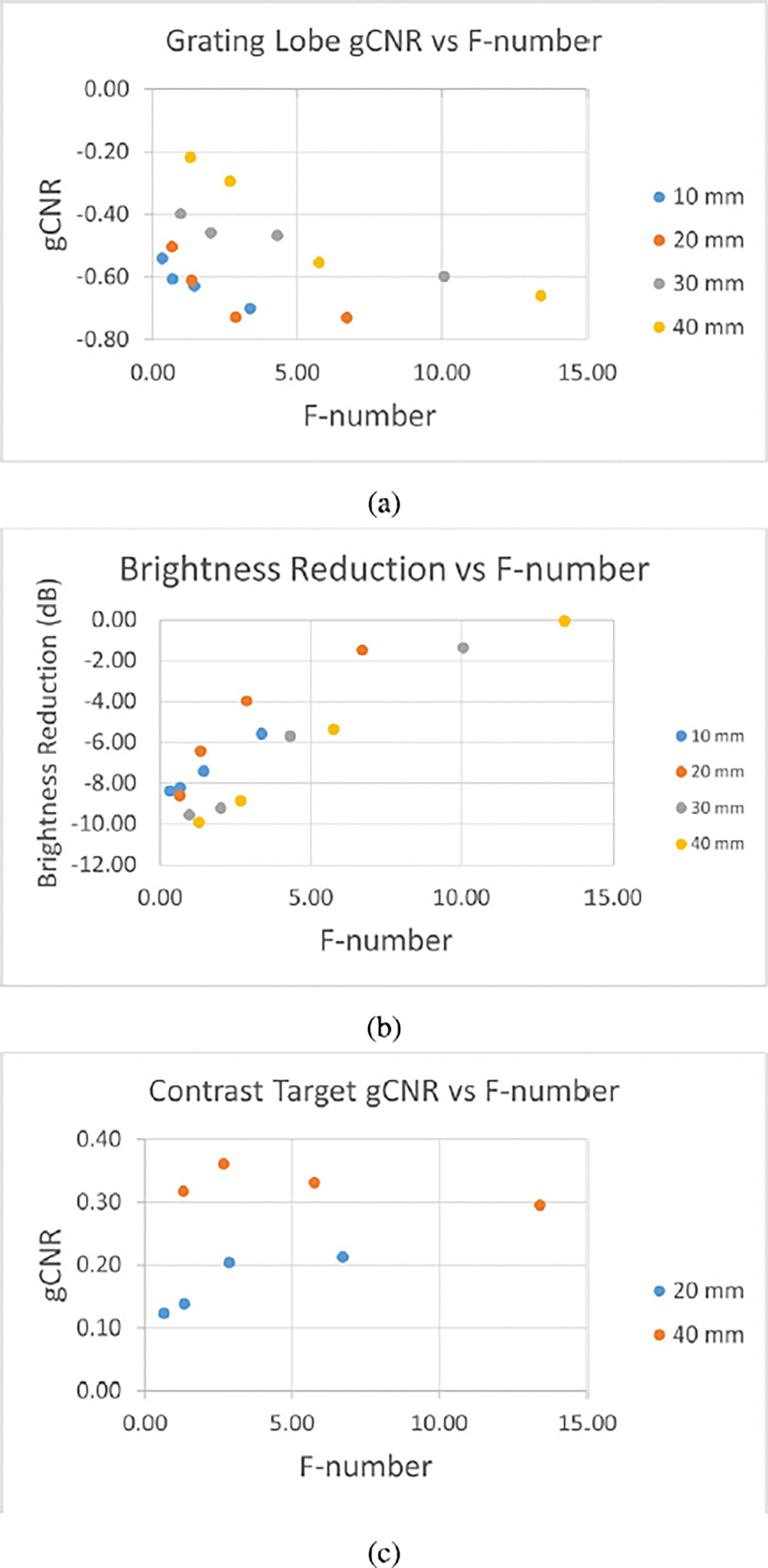
Scatter plots of (a) grating lobe gCNR, (b) grating lobe brightness reduction, and (c) anechoic target gCNR against F-number. Data points are from images beamformed with NSI at a DC offset of 1.

The charts in Fig. 11 provide gCNR and brightness reduction estimates of grating lobes versus the different f-numbers after performing NSI, as well as gCNR of contrast targets versus f-number. Fig. 11(a) shows that for any given depth, contrast of grating lobe regions quickly approaches −1 as f-number increases (i.e. as the sub-aperture size decreases). Similarly, Fig. 11(b) shows major decreases in the amount of grating lobe brightness reduction as f-number increases. Fig. 11(c) shows much greater contrast for the deeper target, especially at the lower f-numbers.

### 3.4. Rabbit abdomen

**Fig. 12.**
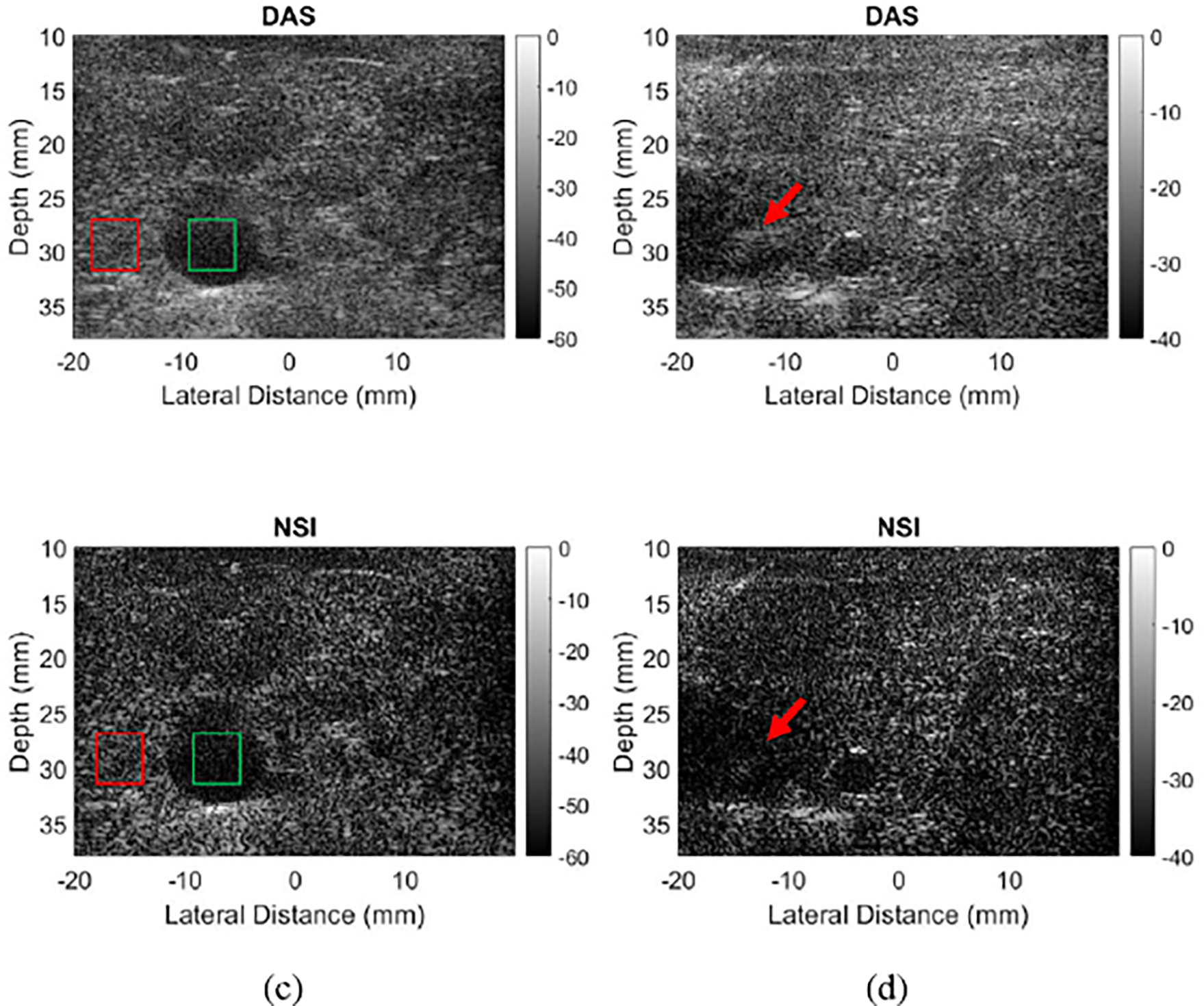
B-mode images of a rabbit liver reconstructed with DAS and NSI. (a) DAS with 2.5λ pitch, the gall bladder gCNR is 0.38. (b) DAS with 5λ pitch. (c) NSI with 2.5λ pitch, the gall bladder gCNR is 0.49. (d) NSI with 5λ pitch. NSI images used a DC offset of 1. Green boxes represent “in” regions for Eq. (7), and red boxes “out” regions. The red arrow points out a grating lobe for the 5λ case.

Fig. 12 shows 4 images of a rabbit liver, two with DAS and two with NSI. Figs. 12(a) and 12(c) show the liver imaged with a 2.5λ pitch with DAS and NSI respectively. The gall bladder is clearly visible in these images as the dark region around −10 mm laterally and 30 mm depth. Fig. 12(c) shows reduced clutter inside the gall bladder compared to Fig. 12(a), with a corresponding increase in gCNR from 0.38 to 0.49. Figs. 12(b) and 12(d) are images taken with a 5λ pitch with DAS and NSI respectively. The gall bladder is again visible in both, as the dark region at −20 mm laterally and 30 mm depth. The circular cross section of a blood vessel is also visible, at around −5 mm laterally and at 30 mm depth. Contrast of the gall bladder slightly increased with NSI, going from a gCNR of 0.50 to 0.56. Contrast of the blood vessel also improved, going from a gCNR of 0.22 to 0.32. Grating lobe artifacts were removed around the bright point marking the top boundary of the blood vessel as well, denoted by the red arrow, with the value of grating lobe brightness reduction reaching −16.2 dB.

## 4. Discussion

### 4.1. Grating lobe reduction versus array pitch

The purpose of varying the pitch size was to quantify the ability of NSI to mitigate grating lobe levels for pitch-to-wavelength ratios greater than one. The results from this experiment can be explained by channel decimation and element factors. Here, the term “element factor” refers to the element sensitivity at different angles, given by the following formula:

(9) $$H\left(\theta_{x}\right)=W\;sinc\left(\frac{Wk_{x}}{2}\right)$$

where $H\left(\theta_{x}\right)$ is the sensitivity, $\theta_{x}$ is the beam direction in the lateral direction, $k_{x}$ is the wave number in the lateral direction, and W is the width of an individual element. As can be inferred from Eq. (9), increasing W increases the argument of the sinc() function, which makes the element factor more narrow. This narrowing of the element factor represents decreased element sensitivity for higher angles. That reduced sensitivity can prevent grating lobes from forming. In the case of Fig. 1, the difference in grating lobe brightness between the left column (2.5λ pitch) and the right column (5λ pitch) is explained by channel decimation creating a higher pitch. That higher pitch brings the grating lobes in closer to the main lobe so that the element factor does not prevent their formation. Thus, much of the grating lobe is still visible in Fig. 1(d), despite being reduced by NSI, and the improvement to gCNR is marginal compared to Fig. 1(c).

Furthermore, in Fig. 3(a), note that the data points at 2.07λ and 3.22λ have very low contrast with DAS and hardly improve with NSI. These data points were made with the L14–5/38 probe, with channel decimation, whereas the data points at 1.63λ and 2.54λ were made with the L14–5/60 probe, without channel decimation. The L14–5/38 probe has smaller elements, so its element factor is wider. The wider element factor, combined with the increased pitch due to channel decimation, meant there were once again visible grating lobe artifacts after NSI, so the gCNR barely improved.

The contrast targets also suffered from channel decimation. Decimating both transmit and receive channels reduced the emitted power, which led to less SNR and penetration. This fact was evident in the images of anechoic targets shown in Fig. 2, and in the data points shown in Fig. 3(b). When the pitch was increased by decimating elements of the aperture, the contrast suffered immensely. However, the improvement to contrast that NSI achieves over DAS was nearly equal, around 0.05–0.1, across all data points in Fig. 3(b). These data suggest that pitch, element factor, and channel decimation all have no effect on NSI’s performance in increasing contrast.

It is also interesting to note that there was little change in the total brightness reduction of grating lobes across all the pitch sizes, shown in Fig. 3(c). In other words, regardless of pitch, NSI with a DC offset of 1 can be expected to provide grating lobe brightness reduction of around 10–15 dB. This result, combined with the rest, suggests that the best performance of NSI on grating lobes is when element factors bring grating lobes to a brightness level of about 10 dB or less above the speckle. Then, NSI will be able to fully eliminate them, leaving no visible artifacts.

### 4.2. Grating lobe reduction versus DC offset

The purpose of this experiment was to quantify the correlation between grating lobe reduction and the DC offset used in NSI. The results demonstrated that lower DC offsets led to greater reduction of grating lobes, displayed most prominently by Fig. 6(c). However, several other factors would prevent a user from setting a very low DC offset, such as 0.1. First, grating lobes appear to be inverted by DC offsets less than 1, blacking out the region the grating lobe used to cover, like in Fig. 8. This observation suggests that for DC offsets less than 1, grating lobe reduction will not always equate to “regained information” or reveal the actual speckle signal. Second, for DC offsets lower than around 0.3, the images tend to be so dark that almost nothing is visible, such as in Figs. 4(c), 4(d), 5(c), and 5(d). This effect is quantified by the sSNR metric, whose trend is shown in Fig. 6(b). While technically the grating lobes are greatly reduced so that their contrast approached zero, the images became so dark that they were not as useful. Thus, the goal for tuning the DC offset on wire targets would be for grating lobe gCNR to be near zero (i.e. the grating lobe was mitigated), while keeping sSNR close to 1.91 (i.e. a visible and useful image). A similar goal can be stated for the contrast targets, where gCNR should be near 1 for the anechoic region, balanced with an sSNR near 1.91.

### 4.3. Grating lobe reduction versus f-number

The purpose of this experiment was to gain a greater understanding of the physical reasons why NSI reduces grating lobes. The results demonstrated that for any given depth, decreasing the f-number resulted in lower gCNR and greater brightness reduction of the grating lobe region. This observation supports what has already been published, that NSI reduces grating lobes due to the broadband nature of ultrasound imaging: only a few elements in the receive sub-aperture contribute to the formation of grating lobes, meaning grating lobes do not experience a true zero-mean apodization. Thus, grating lobes do not form nulls and are eventually subtracted away [16]. Increasing the sub-aperture size would naturally make this effect more pronounced, and therefore lead to greater mitigation of grating lobes.

However, Figs. 11(a) and 11(b) show that for a similar f-number, grating lobes from deeper targets tended to experience greater suppression than shallower targets. Also, the contrast of anechoic regions was much greater for the target at 40 mm than the target at 20 mm in Fig. 11(c). The previously explained behavior of broadband pulses allowing NSI to reduce grating lobes does not change with greater depths, and does not explain these results. Therefore, this experiment suggests that other physical phenomena could be contributing to grating lobe suppression with NSI. The most likely possibility is attenuation, which is a property of the medium that increases with greater depths and with higher frequencies. Given a broadband signal, as the signal gets deeper, the higher frequencies in the signal band are attenuated more than lower frequencies, effectively lowering the center frequency and increasing the wavelength of the pulse. The increased wavelength would naturally reduce grating lobes at greater depths. Then, NSI is more likely to leave no visible grating lobe artifacts, causing greater improvement to gCNR as discussed in Section 4.1.

To quantify the effects of frequency-dependent attenuation on grating lobe reduction, we assumed a Gaussian pulse shape. The L14–5/60 probe used for this experiment had a 60% bandwidth at a center frequency of 7.81 MHz. The CIRS Model 539 Phantom had an attenuation coefficient of 0.5 dB/cm/MHz, and its speed of sound was 1450 m/s. The maximum depth of wire targets used in this experiment was 4 cm. Taking the Fourier Transform of the Gaussian pulse and applying the attenuation for a depth of 4 cm results in a center frequency downshift from 7.81 MHz to a 6.78 MHz. Grating lobe locations are given by θ=arcsin(λ∕d), where θ is the angle of a grating lobe, λ is the wavelength and d is the pitch. Converting our frequency values to wavelengths and plugging them in for λ suggests that grating lobes appear at 23.3 degrees for 7.81 MHz, and 26.9 degrees for 6.78 MHz. Plugging those angles into Eq. (9) yields −37.8 dB at 7.81 MHz and −43.8 dB at 6.78 MHz. Thus, the grating lobes are 6 dB lower at a depth of 4 cm due to the shift of the center frequency.

Note that this experiment, as well as the previous two detailed in Sections 4.1 and 4.2 always aligned wires with the center of the image. This was done without loss of generality, as NSI was still able to produce high resolution and low grating lobes when bright targets were not aligned with the center of the image. NSI was performed across a sliding sub-aperture on receive, so even if a pixel was not in the center of the image, it will still be at the center of the sub-aperture used to beamform it. Thus, echoes from the actual pixel location were lined up with the null formed by the zero-mean aperture, while echoes from other locations (i.e. grating lobes) were not. Therefore, grating lobes will still be subtracted away, even when the targets that formed them were not aligned with the center of the image.

### 4.4. Rabbit abdomen

The purpose of this experiment was to verify phantom results in vivo, using an optimal parameter set suggested from the results of the phantom experiments. Grating lobes mostly appeared around boundaries between liver tissue and blood vessels, and boundaries between the liver and surrounding tissue. The results demonstrated that NSI was able to reduce those grating lobes even for a pitch size of 5λ. However, the bigger concern for abdominal imaging is contrast between different regions such as the liver tissue, gall bladder, and blood vessels. The results indicated that NSI only provided small improvements for contrast.

## 5. Conclusion

This paper has provided the results of several experiments quantifying the ability of NSI to reduce grating lobes. It was found that while NSI was able to achieve dramatic grating lobe reduction at pitch sizes up to 6.5 wavelengths, visible artifacts were left unless NSI was paired with large elements. In this case, the element factor kept grating lobes to around 10 dB above the background in DAS, and NSI completely eliminated grating lobes that DAS had produced. The improvement that NSI offers over DAS for contrast of anechoic regions was marginal but consistent for all pitch sizes, and the best contrast occurred when sufficient channels were used to provide high transmit power and SNR (i.e. when channel decimation was not used). A DC offset of 1 was observed to reduce the grating lobes without much change to the speckle compared to DAS, whereas a DC offset lower than 1 would significantly lower the speckle signal-to-noise ratio and invert the grating lobes. The f-number of the transducer can be lowered by increasing the receive sub-aperture size. The lower f-number will increase the amount of grating lobe reduction at any given depth. Furthermore, a wide receive sub-aperture on a deeper target provided best results for contrast of anechoic regions. Testing NSI on a rabbit abdomen demonstrated that NSI can reduce grating lobes caused by boundaries between blood vessels and tissue, but offers only a small improvement in contrast of large anechoic regions such as blood vessels and the gall bladder. The combination of these results suggests that NSI is much more capable than DAS of producing high quality images on a large-pitch, wide field-of-view array, given that the array has wide enough elements to minimize initial grating lobes, and sufficient channels to provide high transmit power and SNR.

## Figures and Tables

**Table 1:** Pitch size settings. The first column shows which probe was used. The second column shows the frequency of the excitation pulse. The third column shows whether or not channel decimation of 2 was used. The final column represents the estimated pitch size in wavelengths.

Probe	Frequency (MHz)	Channel decimation	Pitch size (wavelengths)

L14-5/60	5	no	1.63
L14-5/38	5	yes	2.07
L14-5/60	7.81	no	2.54
L14-5/38	7.81	yes	3.22
L14-5/60	5	yes	3.26
L14-5/38	10	yes	4.14
L14-5/60	7.81	yes	5.08
L14-5/60	10	yes	6.51

**Table 2:** F-number settings. The first column shows the number of elements in the receive sub-aperture. The second column shows the depth of the target in mm. The final column shows the resulting f-number, which is the ratio of depth in mm over sub-aperture size in mm.

# of elements	depth (mm)	F-number

32	10	0.32
32	20	0.65
16	10	0.67
32	30	0.97
32	40	1.30
16	20	1.34
8	10	1.44
16	30	2.01
16	40	2.68
8	20	2.87
4	10	3.36
8	30	4.31
8	40	5.75
4	20	6.71
4	30	10.07
4	40	13.42

## Data Availability

Data will be made available on request.
